# Interpersonal discrimination and depressive symptomatology: examination of several personality-related characteristics as potential confounders in a racial/ethnic heterogeneous adult sample

**DOI:** 10.1186/1471-2458-13-1084

**Published:** 2013-11-20

**Authors:** Haslyn ER Hunte, Katherine King, Margaret Hicken, Hedwig Lee, Tené T Lewis

**Affiliations:** 1 School of Public Health, Social & Behavioral Sciences, Robert C. Byrd Health Sciences Center, West Virginia University, PO Box 9190, Morgantown, WV 26506-9190, USA; 2 Environmental Public Health Division, U.S. Environmental Protection Agency, 104 Mason Farm Road, Chapel Hill, NC 27514, USA; 3 Department of Epidemiology, University of Michigan, 3634 SPH Tower, 1416 Washington Heights, Ann Arbor, MI 48109, USA; 4 Department of Sociology, University of Washington, Box 353340, 211 Savery Hall, Seattle, WA 98195-3340, USA; 5 Department of Epidemiology, Emory University, 1518 Clifton Rd, NE, CNR Room 3027, Atlanta, GA 30322, USA

**Keywords:** Stress, Psychological, Discrimination (psychology), Depression, Personality

## Abstract

**Background:**

Research suggests that reports of interpersonal discrimination result in poor mental health. Because personality characteristics may either confound or mediate the link between these reports and mental health, there is a need to disentangle its role in order to better understand the nature of discrimination-mental health association. We examined whether hostility, anger repression and expression, pessimism, optimism, and self-esteem served as confounders in the association between perceived interpersonal discrimination and CESD-based depressive symptoms in a race/ethnic heterogeneous probability-based sample of community-dwelling adults.

**Methods:**

We employed a series of ordinary least squares regression analyses to examine the potential confounding effect of hostility, anger repression and expression, pessimism, optimism, and self-esteem between interpersonal discrimination and depressive symptoms.

**Results:**

Hostility, anger repression, pessimism and self-esteem were significant as possible confounders of the relationship between interpersonal discrimination and depressive symptoms, together accounting for approximately 38% of the total association (beta: 0.1892, p < 0.001). However, interpersonal discrimination remained a positive predictor of depressive symptoms (beta: 0.1176, p < 0.001).

**Conclusion:**

As one of the first empirical attempts to examine the potential confounding role of personality characteristics in the association between reports of interpersonal discrimination and mental health, our results suggest that personality-related characteristics may serve as potential confounders. Nevertheless, our results also suggest that, net of these characteristics, reports of interpersonal discrimination are associated with poor mental health.

## Background

Recent reports indicate that roughly nine percent of adults report symptoms of depression, making depression the most common mental illness in the United States (US) [1]. Furthermore, depression is the leading cause of disability for individuals ages 15–44, with an estimated loss of labor productivity exceeding $31 billion per year [2]. Researchers hypothesize that daily stressors may be an important factor in the development of depressive symptoms and disorders [3].

Evidence suggests that perceived interpersonal everyday discrimination is an important type of daily stressor associated with depressive symptoms that may have particular salience for racial/ethnic groups such as African Americans, Hispanics, and Asians [4-11]. In particular, evidence suggests that there is a positive association between reports of interpersonal discrimination and psychological distress, including depressive symptoms [12]. An important limitation in the literature, however, is a lack of clarity on the role of personality-related characteristics in this association. Specifically, it is unclear whether personality-related characteristics mediate or confound the association between interpersonal discrimination and psychological distress.

### Personality-related characteristics, reports of interpersonal discrimination and depressive symptoms

To be considered a confounder, a personality-related characteristic would alter the report of both interpersonal discrimination (due either to exposure or perception) and depressive symptoms [13]. If a confounder, the observed association between interpersonal discrimination and mental health will be biased or altogether spurious. On the other hand, to be considered a mediator, the personality-related characteristic would result (partially or fully) from perceived experiences of interpersonal discrimination and then, in turn, alter the reports of depressive symptoms [13]. While there are some studies that suggest that certain personality-related characteristics (e.g., anger, self-esteem and hostility) increase in response to discriminatory experiences, there is a dearth of empirical examinations on the role of these characteristics in the association between perceived interpersonal discrimination and depressive symptoms. In one study, researchers examined whether personality-related characteristics confounded the association between interpersonal discrimination and depressive symptoms in a sample of 250 gay and bi-sexual men [14]. They found that hostility and neuroticism were both significant confounders of the association between perceived anti-gay discrimination and depressive symptoms, together accounting for 42% of the association. Nevertheless, the interpersonal discrimination measure remained a significant predictor for depressive symptoms [14].

While there is a dearth of evidence specifically on the confounding or mediating role of personality-related characteristics, there is some literature on the separate associations among discrimination, personality-related characteristics, and mental health. For example, research has shown that both anger repression (inward reactions or behaviors when experiencing feelings of anger) and anger expression (reactions or behaviors towards others when experiencing feelings of anger) are related to both reports of interpersonal discrimination and depressive symptoms [15-24]. While it may be that perceived interpersonal discrimination results in anger, suggesting a mediating role, results from other research have reported that the perceived expression of anger can serve to encourage or prompt discriminatory behavior from others, suggesting a confounding role [25].

Cynical hostility, a personality-related characteristic denoted by general cynicism and mistrust [26], has been shown to be associated with both perceived interpersonal discrimination [14] and depressive symptoms [27-29]. Research has shown that hostility may be a response to perceptions of interpersonal discrimination suggesting a mediating role [16,23]. However, like anger, hostility may elicit discriminatory responses from others suggesting a confounding role [14]. In fact, research has shown that individuals with high levels of hostility report more suspicious and fewer rational thoughts in reaction to interpersonal scenarios compared to those with low levels of hostility [30]. Similarly, a person with negative affect, such as high hostility, may erroneously recollect past experiences as discriminatory, even though these experiences would not be assessed similarly by a person without negative affect [31].

Likewise, the role of optimism and pessimism is not entirely clear base don the existing literature. Low levels of optimism, which is the generalized expectancy that one will experience good outcomes in the future [27], has been associated with a higher likelihood of interpreting an interpersonal event as discriminatory [9,28]. Similarly, high levels of pessimism have been associated with a higher likelihood of interpreting a negative interpersonal event as discriminatory [29]. This suggests that optimism and pessimism may have confounding roles in the association between reports of interpersonal discrimination and depressive symptoms [32-34]. On the other hand, some research suggests that stressful situations that are interpreted as uncontrollable [35-38] may decrease optimism and increase pessimism and subsequently increase depression [32]. Interpersonal discrimination may be viewed as an uncontrollable form of stress [15,16], which would imply a mediating role for optimism and pessimism.

Self-esteem, defined as a person’s overall sense of self-worth or personal value, has been linked with both reports of interpersonal discrimination and depressive symptoms in many [24,36-42] but not all studies [43,44]. While there is debate in the literature on the causal direction of the discrimination-self-esteem association [44], it may be that some groups of individuals who experience interpersonal discrimination internalize some of the negative social devaluation of their group leading to lowered self-esteem [45] (and other poor mental health outcomes [46-49]), suggesting a mediating role. On the other hand, other study findings suggest that, similar to optimism, higher self-esteem is associated with lower likelihoods of perceiving interpersonal discrimination [50] suggesting a confounding role.

In order to better understand the association between reports of interpersonal discrimination and mental health, research is needed to clarify the mediating and/or confounding role of personality-related characteristics in this association [14,51,52]. Internalized discrimination, which is a self-defeatist mindset developed from the internalization of negative messages of a particular group membership, may result from interpersonal discriminatory experiences. Researchers have argued that this internalized discrimination may be associated with certain personality-related characteristics, such as low self-esteem and periodic states of anger. However, it may be that adverse social conditions result in certain personality-related characteristics that may, in turn, result in more reports of interpersonal discrimination. For example, Williams [53] suggests that the high levels of material deprivation found in some racially- or ethnically-segregated communities are often associated with factors (e.g. low socioeconomic status (SES), poor quality medical care, etc.) that may promote high levels of low self-esteem and anger, which subsequently may lead to reports of perceived interpersonal discrimination. In other words, contextual factors at the neighborhood or community level may influence the development of personality-related characteristics in ways that impact the perception of interpersonal discrimination or increase the likelihood that others will act in discriminatory ways. Taken together, this body of research suggests that personality-related characteristics might serve as both mediators and confounders in the association between interpersonal discrimination and mental health.

In this study we chose *a priori* to examine the potential confounding (rather than the mediating) role of several personality-related characteristics in the association between perceived experiences of interpersonal discrimination and depressive symptoms. This approach was selected for two main reasons. First, as with the existing literature, the data we use is cross-sectional in nature, making it impossible to statistically distinguish between a confounding and mediating role. Indeed, it is likely that ***both*** mediation and confounding may be at play in some type of feedback (cross-lagged) loop for some, if not all, of the personality-related characteristics we examined. Second, critics of the perceived interpersonal discrimination measures may argue that these measures do not reflect actual experiences of discrimination, but simply the psychological characteristics of the respondent. This would imply that the psychological characteristics, not the perceptions of discrimination, are really harmful for mental health.

We examined the extent to which several personality-related characteristics (hostility, anger repression and expression, pessimism, optimism and self-esteem), collectively served to possibly confound the association between perceived interpersonal discrimination and depressive symptoms in a racial/ethnic heterogeneous probability-based sample of community-dwelling adults. We hypothesized that: (1) personality-related characteristics would possibly confound the association between perceived interpersonal discrimination and depressive symptoms; and (2) perceived interpersonal discrimination would remain significantly associated with depressive symptoms after adjusting for these potential confounders.

## Methods

### Sample

The Chicago Community Adult Health Study (CCAHS) is a stratified, multi-stage probability sample of 3,105 adults aged 18 years and over, living in Chicago, Illinois [54]. Briefly, the CCAHS, conducted between May 2001 and March 2003, obtained both self-reported and objective measures of individual and household psychosocial risk factors and resources, social factors, and residential contexts. The CCAHS sample includes 802 Hispanics, 1240 non-Hispanic Blacks (Blacks), 983 non-Hispanic Whites (Whites), and 80 individuals of other races/ethnicities. One adult per household was interviewed face-to-face, with a response rate of 71.82%. The weighted sample matched the distribution of the 2000 Census population estimates for the city of Chicago in age, race/ethnicity and sex. Additional study details are provided elsewhere [54].

### Measures

Depressive symptoms were measured using the 11-item version of the Center for Epidemiologic Studies Depression Scale (CESD) [55]. The CESD has been validated across various groups including by race and gender [52,53,56]. Participants were asked the frequency, in the previous two weeks, with which they experienced certain feelings (e.g. hopelessness, restless sleep, loneliness, fear, sadness and changes in appetite). Responses were provided on a Likert-like scale of 1(never) to 4 (most of the time). A scale was created as the sum of the responses, divided by 11, for a range of 1.00 to 3.82 (Cronbach’s α = 0.85).

The perceived everyday discrimination scale, a measure of perceived day-to-day interpersonal discrimination, assesses the occurrence and frequency with which individuals encounter routine and relatively minor experiences of unfair treatment [57]. In this study, participants were asked how often in their lifetime (1) they were treated with less courtesy or respect than others, (2) they received poorer service than others, (3) they believed others acted as if they were not smart, (4) others acted as if they were afraid of them, or (5) they felt threatened or harassed. Responses were provided on a Likert-like scale of 1(at least once a week) to 5 (never). A scale was created as the sum of the reverse-coded responses, divided by 5, for a range of 0 (no perceived discrimination) to 4 (highest level of perceived discrimination) (Cronbach’s α = 0.75).

Cynical hostility was measured using a modified 5-item version of the Cook-Medley cynical hostility scale [58]. Participants were asked the extent to which they agreed with the following statements: 1) most people inwardly dislike putting themselves out to help other people; 2) most people will use somewhat unfair means to gain profit or an advantage rather than lose it; 3) no one cares much what happens to you; 4) I think most people would lie in order to get ahead; 5) I commonly wonder what hidden reasons another person may have for doing something nice for me. Responses were provided on a Likert-like scale of 1 (agree strongly) to 4 (disagree strongly). The scale (Cronbach’s α = 0.74) was created with the mean of the five statements resulting in a range of values of 1 (lowest level of cynical hostility) to 4 (highest level of cynical hostility).

Expressed and repressed anger were measured using an abridged version of Spielberger’s anger-out and anger-in expression scales, respectively [59]. Participants were asked the frequency of specific typical reactions or behaviors when they are angry or mad. Expressed anger reactions and behaviors included: arguing with others, striking out, saying nasty things, and losing temper. Repressed anger reactions and behaviors include: keeping things in, withdrawing from people, getting irritated more than people are aware, and getting angrier more than willing to admit. Responses were provided on a Likert-like scale of 1 (almost never) to 4 (almost always). The anger-in scale (Cronbach’s α = 0.71), constructed by taking the mean of the values for each of the four statements, had a range of 1 (lowest level of anger-In) to 4 (highest level of anger-In). The anger-out scale (Cronbach’s α = 0.76), constructed by taking the mean of the values of the four anger-out statements, had a range of 1 (lowest level of anger-out) to 4 (highest level of anger-out).

Three items from the Life Orientation Test–Revised [60], was used to form a scale that assesses dispositional optimism and pessimism (Cronbach’s α = 0.73). A sample item is “In uncertain times, I usually expect the best.” Participants rated the extent of their agreement with each item on a Likert-type scale ranging from 1 (strongly disagree) to 5 (strongly agree).

We used an abridged 4-item version of the Rosenberg Self-Esteem scale [61]. Participants reported the extent to which they agreed with statements about their positive attitude toward themselves, satisfaction with themselves, feelings of usefulness and overall goodness. Responses were provided on a Likert-like scale of 1 (disagree strongly) to 4 (agree strongly). The range of values for the self-esteem scale (Cronbach’s α = 0.71) is from 1 (lowest level of self-esteem) to 4 (highest level of self-esteem).

Covariates included age, sex, race/ethnicity (Hispanics, non-Hispanic Blacks, non-Hispanic Others and non-Hispanic Whites), annual household income category ($4,000, $5,000-$9,999, $10,000-$29,999, $30,000-$49,999 and ≥ $50,000), lifetime education status (< 12 years, 12 years, and > 12 years), employment status (employed and not currently employed), marital status (married and not currently married), nativity status (US and foreign born), a count of self-reported chronic health conditions (ranging from 0–9) and a count of stressful major adverse life events (0–11).

### Data analyses

The analytic aim of the paper was to determine whether and how much the selected personality-related variables potentially confounded the relationship between interpersonal discrimination and depressive symptoms. We first estimated means with standard errors of continuous variables and percentages within categorical variables in our total sample (see Table 1). We then examined the first-order correlation between perceived interpersonal discrimination and the personality-related characteristics (see Table 2).

**Table 1:** Demographic characteristics of participants in the Chicago community adult health study, 2001–03 (N = 3,105)

	**No.**	**%**	**Mean, (SE)**
Age, years			42.47 (0.42)
Sex			
Male	1,471	47.38%	
Female	1,634	52.62%	
Race/Ethnicity			
Hispanics	801	25.81%	
Non-Hispanic Whites	1,191	38.36%	
Non-Hispanic Blacks	996	32.07%	
Non-Hispanic Others	117	3.77%	
Annual Household Income			
0-4 K	280	9.02%	
5 K-9 K	234	7.55%	
10 K-29 K	860	27.68%	
30 K-49 K	648	20.88%	
50 K+	1083	34.86%	
Educational Status			
< 12 years	727	23.42%	
12 years	738	23.75%	
> 12 years	1,640	52.83%	
Employment Status			
Not Employed	1,107	35.64%	
Employed	1,998	64.36%	
Marital Status			
Not Married	1,807	58.19%	
Married	1,298	41.81%	
Nativity Status			
Foreign Born	835	26.89%	
Born in US	2,270	73.11%	
Major Stress			2.36 (0.04)
Chronic Illness			1.13 (0.04)

*Abbreviations*: *SE* standard error, *US* United States.

**Table 2:** Means, Standard Deviations (SD), Coefficient Alphas (α) and Pearson correlations of the personality-related measures

**Measures**	**Mean**	**S.D.**	**1**	**2**	**3**	**4**	**5**	**6**	**7**
1. CESD	1.86	0.58	___						
2. Discrimination	0.75	0.78	−0.309***	___					
3. Hostility	2.56	0.62	−0.323***	−0.207***	___				
4. Anger repression	2.12	0.67	−0.318***	−0.170***	−0.176***	___			
5. Anger expression	1.61	0.58	−0.200***	−0.202***	−0.131***	−0.230***	___		
6. Pessimism	1.96	0.77	−0.373***	−0.070***	−0.402***	−0.146***	−0.119***	___	
7. Optimism	3.27	0.63	−0.205***	−0.081***	−0.002***	−0.107***	−0.117***	−0.185***	___
8. Self-esteem	3.40	0.59	−0.466***	−0.143***	−0.211***	−0.222***	−0.216***	−0.460***	0.384***

Note: ***p < 0.001, two tailed.

We used multivariable ordinary least squares (OLS) regression analyses to estimate the confounding role of the personality-related characteristics in a series of models, controlling for the covariates shown in Table 1 (see Table 3). First, we examined the total effect of perceived interpersonal discrimination on depressive symptoms controlling for the covariates (path c). We then separately characterized the association between perceived interpersonal discrimination and each of the personality-related characteristics as the outcome (path α). Although seemingly counterintuitive at first glance, as MacKinnon [13] noted, the proper regression models to test for possible confounding require that the potential confounder (i.e., personality-related characteristics) be modeled as an outcome of the predictor (i.e., perceived interpersonal discrimination) [13]. Next, we examined the effect of interpersonal discrimination on depressive symptoms (path β), controlling for all of the personality-related characteristics. We used the Sobel method [62], which divides the product of nonstandardized coefficients for path α and path β by the by the standard error of this product, to determine if the personality-related characteristics were statistically significant in their presumed role as potential confounders. Lastly, we modeled the effect of perceived interpersonal discrimination on depressive symptoms (path c’) controlling for all of the personality-related characteristics. Informed by previous investigations, multiplicative interaction terms between the interpersonal discrimination variables and race/ethnicity were explored in the multivariable analyses; however, because none were significant, the multivariable analyses included race/ethnicity as a covariate.

**Table 3:** **Unstandardized regression coefficients**^**1 **^**from OLS regression predicting CESD**^**2 **^**using personality-related characteristics (n = 3,105)**

**Measures**	**α paths]** **Disc < = Conf.**	**β paths** **Conf. = > CESD**	**α paths x β paths** **Indirect effect**	**Sobel test statistic and SE for indirect effect**	**Proportion confounded**
Hostility	−0.1537**	−0.1072**	0.0165	−4.72, 0.004**	0.0871
Anger-in	−0.0985**	−0.1098**	0.0108	−3.78, 0.003**	0.0572
Anger-out	−0.1314**	−0.0226	0.0030	−1.28, 0.002	0.0157
Pessimism	−0.1081**	−0.0746**	0.0081	−3.56, 0.002**	0.0426
Optimism	−0.0701**	−0.0393*	0.0028	−1.96, 0.001~	0.0146
Self-esteem	−0.1121**	−0.2722**	0.0305	−5.48, 0.006**	0.1613
		Total indirect effect		0.0716	
		Direct effect		0.1176	
		Total effect		0.1892	
		Total proportion confounded		0.3784	

^1^Controlling for age, sex, income, education, race/ethnicity, employment status, marital status, US nativity status, chronic illness and experiences of stressful life events.

^2^Center for Epidemiologic Studies Depression Scale (CESD).

*Abbreviations*: *Disc* Average Daily Discrimination, *Conf.* Confounding variable.

~p < 0.06, *p < 0.05, **p < 0.001.

Although likely missing at random, missing data were imputed using an iterative method that imputes multiple variables by using chained equations, a sequence of univariate imputation methods with fully conditional specification of prediction equations [63]. The final sample post-imputation for this investigation consisted of 3,105 respondents. All analyses were weighted to account for non-response and complex survey design. With the exception of the correlation analyses presented in Table 2, all of the analyses used the imputed data. All of the analyses were conducted using STATA (v12.1, Stata Corp., 2011).

## Results

The weighted sociodemographic characteristics are presented in Table 1. Respondents were on average 43 years of age, with an age range of 18–92. Approximately half of the sample was male, 26% Hispanic, 38% White, 32% Black and 3.8% of another racial/ethnic group. The average educational level was approximately 13 years (53% reporting more than 12 years of education) and approximately 44% of the sample reported less than $30,000 in annual household income.

Table 2 shows the means, standard deviations, and correlation coefficients for the perceived interpersonal discrimination, depressive symptoms and personality-related characteristics. The mean CESD and interpersonal discrimination scores were 1.86 and 0.75, respectively; these measures were positively correlated. Both perceived interpersonal discrimination and depressive symptoms were positively correlated with the personality-related characteristics, with the exception of optimism and self-esteem which were negatively correlated with the depression symptoms and interpersonal discrimination variables.

**Figure 1:**
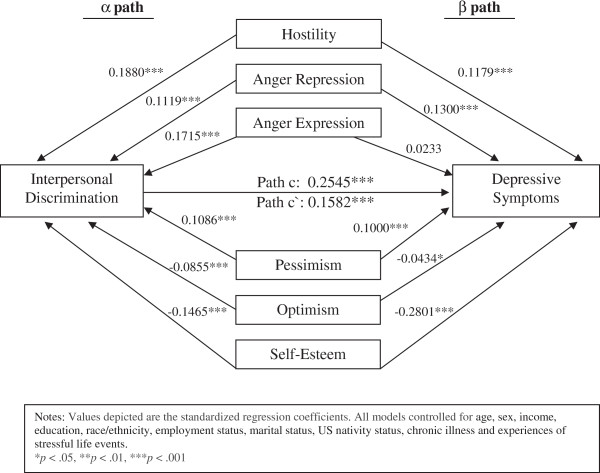
Potential confounding effects of the personality-related characteristics on the relation between perceived interpersonal discrimination and depressive symptoms.

Standardized coefficients representing the personality-related characteristics from the regression models were used to construct the path model depicted in Figure 1. Controlling for the covariates in Table 1, perceived interpersonal discrimination positively predicted depressive symptoms (path c = 0.2545, p < 0.001). However, after controlling for the potential confounding role of the personality-related characteristics, the magnitude of the association between interpersonal discrimination and depressive symptoms was reduced but not eliminated (path c’ = 0.1582, p < 0.001). With the exception of the anger expression-depressive symptoms association, all of the personality-related characteristics were statistically related to both interpersonal discrimination and depressive symptoms (p < 0.05). Individuals who reported high levels of hostility, anger repression, and pessimism were more likely to report higher levels of depressive symptoms and higher levels of interpersonal discrimination than individuals who reported lower levels of these personality-related characteristics. In contrast, individuals with higher levels of optimism and self-esteem were more likely to report lower levels of interpersonal discrimination and depressive symptoms than those who reported lower levels of optimism and self-esteem. Following self-esteem (standardized beta = −0.2801, p < 0.001), interpersonal discrimination was the largest predictor of depressive symptoms after controlling for the potentially confounding effects of the personality-related characteristics.

Table 3 presents the unstandardized regression coefficients, the Sobel test statistics, standard errors, and p-values, for each of the personality-related characteristics illustrating the hypothesized individual and total potential confounding role. Results from the Sobel test suggest that hostility, anger repression, pessimism, and self-esteem were all possible confounders (p < 0.001). The overall indirect effect of the personality-related characteristics was 0.0716, accounting for approximately 38% of the original association between perceived interpersonal discrimination and depressive symptoms.

## Discussion

We examined the role of several personality-related characteristics as potential confounders between perceived interpersonal discrimination and depressive symptoms in a racially/ethnically-heterogeneous, probability-based sample of community-dwelling adults. Although some of these personality-related characteristics were potential confounders, the association between interpersonal discrimination and depression symptoms persisted. While others have examined separate pieces of the associations among perceived interpersonal discrimination, various personality characteristics, and depression, this study is one of the first to characterize the possible confounding role of more than two personality-related characteristics. An additional strength of this study is the generalizability of the results, as we used a representative, multiethnic sample of community dwelling adults, unlike previous studies.

Overall, the findings of this study are consistent with this literature in several ways. First, higher levels of perceived interpersonal discrimination were associated with increased depressive symptoms, controlling for all of the personality-related characteristics and additional covariates. Notably, perceived interpersonal discrimination was the second largest predictor of depressive symptoms after self-esteem, when controlling for the other personality-related characteristics [4-11]. Second, increased levels of hostility, the outward expression of anger, and pessimism were all positively related to higher levels of depressive symptoms [64]. Third, high levels of hostility [14] and the outward expression of anger [19,65] predicted high levels of perceived interpersonal discrimination.

Although researchers suggest that personality may confound the association between perceived interpersonal discrimination and mental health, there is a dearth of empirical work in this area. In one study, Huebner and his colleagues [14] showed that anti-gay interpersonal discrimination was associated with depression after controlling for the potential confounding effects of hostility and neuroticism. Along with our results, these findings suggest that some personality-related characteristics may indeed be antecedent to interpersonal discrimination. Nevertheless, perceived interpersonal discrimination remained an important risk factor for depressive symptoms.

The stability of reports of discrimination over long periods of time might lend support for the notion that personality-related characteristics are subsequent to reports of interpersonal discrimination, and thus play a mediating role. The literature on reports of interpersonal discrimination over time is limited, as there are few datasets with this information at more than one time point. However, the small literature suggests that there is some stability in reports for a large portion of the population. For example, using data from the 1995–2005 cohort of the Midlife Development in the United States, researchers showed that approximately one quarter of the sample reported each of the possible trends over time: consistently low levels, consistently high levels, a decrease, or an increase in perceived interpersonal discrimination [66]. In a study of multiracial/ethnic, middle-aged women, reports of perceived interpersonal discrimination in the past 12 months were relatively stable (range of the within-person stability varied from 0.85 to 0.91) over the course of four to five years [67]. Stability in reports of interpersonal discrimination could be due stability in exposure (i.e. people live, work, play in the same environments from year to year). Alternatively, there may be stability in the perceptions of interpersonal discrimination, which might suggest that these perceptions are more influenced by personality characteristics than originally hypothesized in the existing literature.

Although this study is one of the first to examine the potential confounding role of personality-related characteristics, it is not without limitations. First, to reduce the respondents’ response burden, abbreviated scales of the psychometric measures were employed. Although the reliability of some of the measures may have been reduced, the correlations between measures were strong.

Second, our data were cross-sectional, as has been the case for most empirical studies in this area. Many of the personality-related characteristics we examined are not only correlated with depressive symptoms and interpersonal discrimination, but potentially causally linked through mechanisms not fully understood. It is not fully clear whether the personality-related characteristics are mediators or confounders. In line with previous research, we *a priori* conducted our analyses to determine whether the personality-related characteristics were antecedents to perceived interpersonal discrimination [14,50]; however, because interpersonal discrimination may in fact be antecedent to the personality-related characteristics [19,68-71], they may be mediators and not confounders. Because confounding and mediation effects in cross-sectional data are generally estimated with the same statistical methods, the association of interest can only be distinguished on conceptual or theoretical grounds [13]. Despite the fact that some studies suggest that interpersonal discrimination is antecedent to various personality-related characteristics (implying mediation and not confounding), most of these studies use cross-sectional data, exposing them to the same limitation [19,40-42,44,64,70,72-78].

We are aware of only two studies that have used longitudinal data to disentangle the directionality of these associations [19,68]. In a diary study with 113 adults that collected entries over a one-day period, Broudy et al. [19] showed that baseline measures of ethnic interpersonal discrimination were positively associated with daily levels of anger. Similarly, results based on a longitudinal sample of North American indigenous adolescents, perceived interpersonal discrimination was associated with increased anger over a longer period of time [68]. However, we are not aware of any studies that have specifically examined the potential mediating-confounding association of personality-related characteristics with perceived interpersonal discrimination and depressive symptoms over time. Indeed, it is likely that personality-related characteristics serve as both mediators and confounders in this association.

Future studies utilizing longitudinal data, along with methods that can address potential cycling or cross-lagged effects (e.g., perceived interpersonal discrimination influencing hostility which in turn influences perceived interpersonal discrimination which furthers influences hostility, and so on) are warranted. Because some personality-related characteristics, both positive and negative, may change over time, future research in this area must pay special attention to these methodological issues.

## Conclusion

This study is among the first to empirically characterize the potential confounding role of multiple personality-related characteristics simultaneously in the association between perceived interpersonal discrimination and depressive symptoms in a representative, multi-ethnic sample of community dwelling adults. Our results suggest that personality-related characteristics potentially confound this association. Nevertheless, perceived interpersonal discrimination remained significantly associated with depressive symptoms after adjusting for these potential confounders, supporting results from population-based studies that have consistently showed that perceived interpersonal discrimination is deleterious to an individual’s health and well-being [52]. In order to clarify the confounding versus mediating role of personality-related characteristics, future research using longitudinal data and sophisticated methods to disentangle the causal association between perceived interpersonal discrimination and mental health over time is warranted.

## Competing interests

The authors declare that they have no competing interests.

## Authors’ contributions

HERH conducted the study design study analysis. HERH, KK, MH and HL drafted portions of the manuscript. All authors revised and approved the final manuscript.

## Pre-publication history

The pre-publication history for this paper can be accessed here:

http://www.biomedcentral.com/1471-2458/13/1084/prepub
